# Reactive oxygen species (ROS)-responsive nanoprobe for bioimaging and targeting therapy of osteoarthritis

**DOI:** 10.1186/s12951-021-01136-4

**Published:** 2021-11-27

**Authors:** Chong Shen, Ming Gao, Haimin Chen, Yanting Zhan, Qiumei Lan, Zhimin Li, Wei Xiong, Zainen Qin, Li Zheng, Jinmin Zhao

**Affiliations:** 1 Guangxi Engineering Center in Biomedical Materials for Tissue and Organ Regeneration, The First Affiliated Hospital of Guangxi Medical University, Nanning, 530021 China; 2 Department of Orthopedics, The Affiliated Hospital of Guilin Medical University, No. 15 Lequn Road, Guilin, 541001 Guangxi China; 3 Guangxi Collaborative Innovation Center for Biomedicine, The First Affiliated Hospital of Guangxi Medical University, Nanning, 530021 China; 4 Department of Orthopaedics Trauma and Hand Surgery, The First Affiliated Hospital of Guangxi Medical University, Nanning, 530021 China; 5 Guangxi Key Laboratory of Regenerative Medicine, International Joint Laboratory On Regeneration of Bone and Soft Tissue, The First Affiliated Hospital of Guangxi Medical University, Nanning, 530021 China

**Keywords:** ROS-responsive, Drug delivery, Targeting therapy, Osteoarthritis

## Abstract

Stimulus-responsive therapy that allows precise imaging-guided therapy is limited for osteoarthritis (OA) therapy due to the selection of proper physiological markers as stimulus. Based on that the over-production of Reactive Oxygen Species (ROS) is associated with the progression in OA, we selected ROS as markers and designed a cartilage targeting and ROS-responsive theranostic nanoprobe that can be used for effective bioimaging and therapy of OA. This nanoprobe was fabricated by using PEG micelles modified with ROS-sensitive thioketal linkers (TK) and cartilage-targeting peptide, termed TKCP, which was then encapsulated with Dexamethasone (DEX) to form TKCP@DEX nanoparticles. Results showed that the nanoprobe can smartly “turn on” in response to excessive ROS and “turn off” in the normal joint. By applying different doses of ROS inducer and ROS inhibitor, this nanoprobe can emit ROS-dependent fluorescence according to the degree of OA severity, helpful to precise disease classification in clinic. Specifically targeting cartilage, TKCP@DEX could effectively respond to ROS and sustained release DEX to remarkably reduce cartilage damage in the OA joints. This smart, sensitive and endogenously activated ROS-responsive nanoprobe is promising for OA theranostics.

**Supplementary Information:**

The online version contains supplementary material available at 10.1186/s12951-021-01136-4.

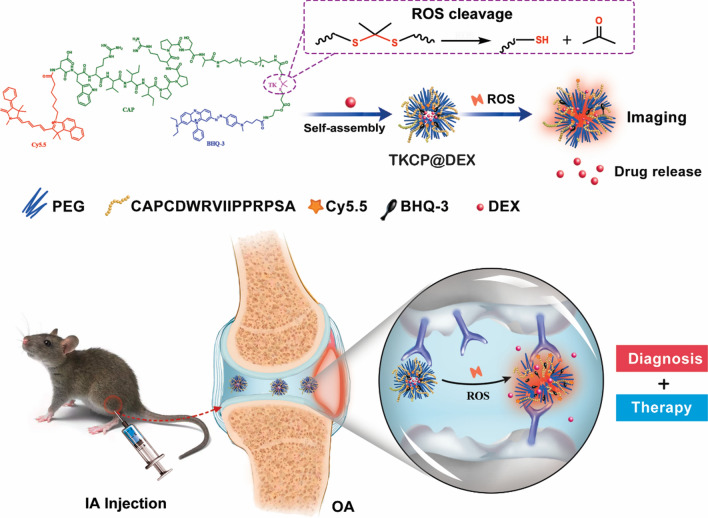

## Introduction

Osteoarthritis (OA) is a chronic disease characterized by the progressive degeneration of cartilage that leads to joint pain and even serious disabilities of patients around the world [1, 2]. At the end-stage of OA, the joints gradually lose function and need prosthetic replacements surgery [3]. Thus, it is necessary to highlight the diagnosis and treatment of OA.

Nowadays, endogenous-based fluorescent probes relying on certain physiological markers like NO [4] and MMP-13 [5–8] to generate fluorescence have been explored for detection of OA in vivo. However, these probes depending on pathological events are limited in clinical applications mainly due to the relatively low concentrations of endogenous biochemical markers [9–12], resulting in inaccurate and insensitive signals in the deep tissues. Moreover, most probes can only be used for monitoring and have little or no therapeutic effects on OA. Therefore, it is imperative to find remarkable physiological markers and design a responsive theranostic probe that can penetrate through the deep tissues and simultaneously have therapeutic potential for OA.

It is generally accepted that Reactive Oxygen Species (ROS) are important causative factors during the development of OA. It has been reported that the levels of ROS, e.g., H_2_O_2_, O_2_^−^, HO^−^, and HOCl, which maintain at a low level in normal articulatory [13], are dramatically increased (may up to 50–100 μM) in the joints of OA patients [14, 15]. The over-generation of ROS elicits hyper-peroxidation, protein carbonylation and DNA damage, which has been considered as the main mechanism of cartilage cells loss and tissue damage [16]. But until now, a specific and biocompatible ROS-responsive system has not yet been moved forward to real-time monitoring and therapy of OA.

The drug delivery systems based on ROS-responsive functional moieties such as sulfide [17], phenylboronic acids and esters [18–20], selenium-containing linkage [21], peroxalate [22–24] etc., have been widely used for cancer treatment. Among various materials, nano-scaled ROS-responsive polyethylene glycol (PEG) polymers composed of thioketal linkers attracted most intention because of their sensitivity and responsiveness to endogenous ROS down to submicromolar concentrations (~ 100 μΜ) at deep physiological signaling levels [25–27]. PEG is usually employed to self-assemble micelles due to their excellent biocompatibility, which can shield from the mononuclear phagocyte system (MPS) and prevent host rejection after injected in vivo [28]. Moreover, the PEG micelles of sufficiently small size from 20 to 200 nm facilitates entry into the dense cartilage. Further modification with a targeted biomolecular ligand may resist rapid clearance from the joint site [29, 30]. Thus, the ROS-responsive PEG micelles based on thioketal linkers may hold promise for OA treatment in clinical.

**Scheme 1:**
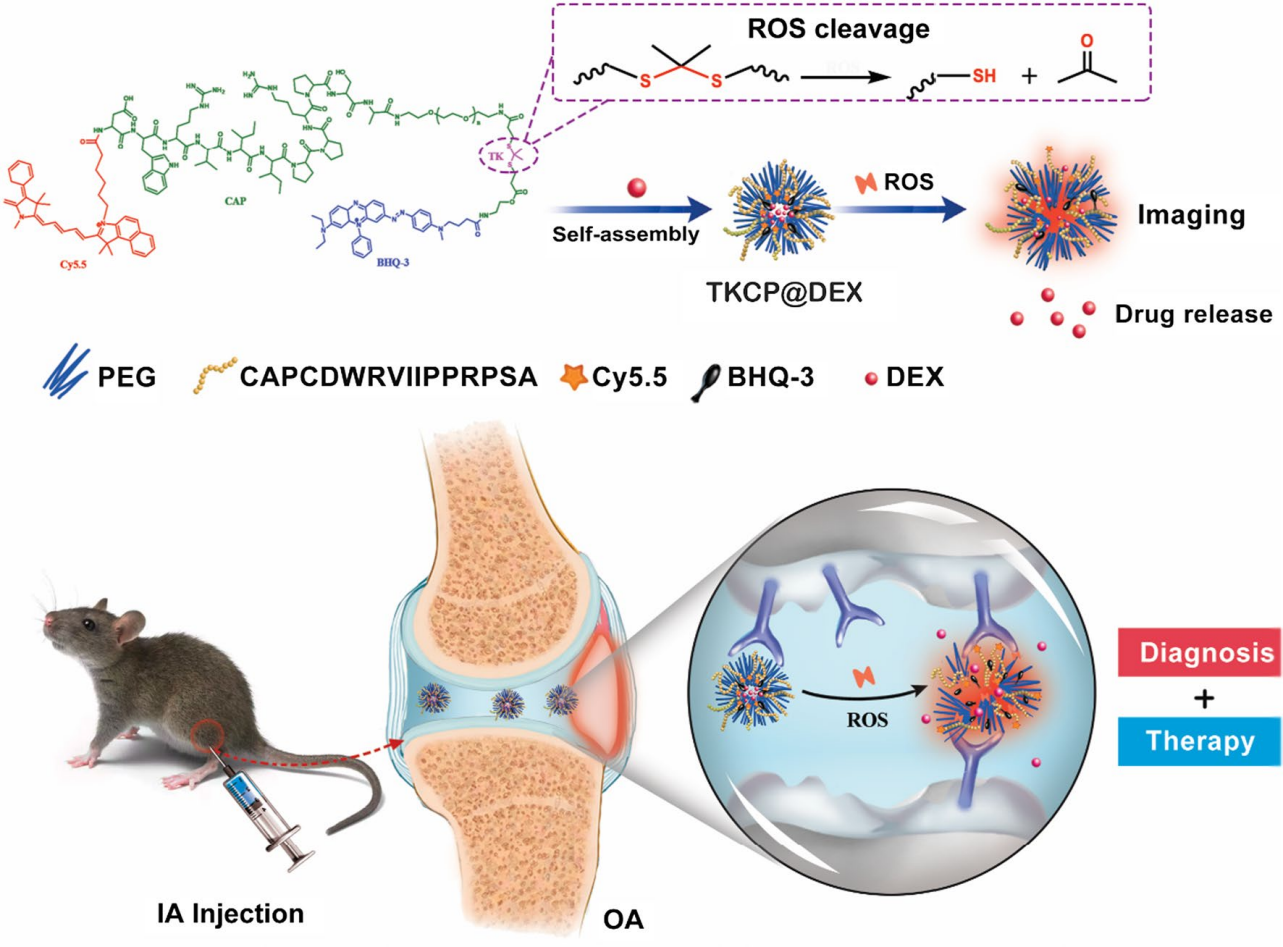
Schematic illustration of the self-assembly of ROS-responsive nanoparticles for bioimaging and targeted therapy of osteoarthritis in vivo

Herein, we developed a multifunctional ROS-activatable theranostic polymer nanoparticles that are capable of loading hydrophobic drug and self-reporting the payload release upon ROS stimulation. The nanoparticles are formed by the amphiphilic block copolymers consisting of Cy5.5 modified cartilage-targeting peptide (CAP, DWRVIIPPRPSA) [31, 32] and PEG modified an oxidation-responsive thioketal linkers (TK) hydrophobic block that contains Black Hole Quencher 3 (BHQ-3) as a quencher for Cy5.5, which was then encapsulated with Dexamethasone (DEX) to form TKCP@DEX nanoparticles. DEX is a broad-spectrum synthetic corticosteroid medicine with long-lasting anti-inflammatory effect to potently reduce glycosaminoglycan loss in OA-affected cartilage site [33–35]. As show in Scheme 1, the smart TKCP@DEX nanoparticles specifically target on articular cartilage by CAP and respond to the high level of ROS since the thioketal linkages were cleaved by abundant ROS in inflamed tissues, leading to gradual disassembly of the polymer to release Cy5.5 and drug. The increased distance between quencher (BHQ-3) and Cy5.5 enables stronger fluorescence signal from Cy5.5, providing effective monitoring of the progression of OA. But in normal condition where ROS are minimal, the fluorescence is turned off by BHQ-3. This smart cartilage-targeting ROS responsive theranostic nanoprobe is promising for OA therapy.

## Materials and methods

### Synthesis of ROS-responsive polymer (TKCP)

Here, thioketal were synthesized via their reaction with 3-mercaptopropionic acid and acetone. The fluorescent monomer modified polyethylene glycol (Cy5.5-CAP-PEG) was synthesized following a route as shown in Additional file 1: Figs. S1 and S3. BHQ-3 was chosen as a quencher and co-polymerized with a ROS-cleavable thioketal-containing linker to prepare the Cy5.5-CAP-PEG-TK-BHQ-3 (TKCP) (Additional file 1: Figs. S2, S3). For comparison, we also synthesized TKP without cartilage-targeting peptide (CAP) and CAPP without thioketal groups as controls (Additional file 1: Figs. S4, S5). The detailed preparation protocols of ROS-responsive polymers are presented in the Additional file 1 and verified by ^1^H NMR spectra.

### DEX loading

20 mg of nanoparticles was dispersed in 0.5 mL tetrahydrofuran (THF), followed by the addition of 10 mg DEX (dissolved in 0.5 mL tetrahydrofuran). Then 10 mL deionized water with a syringe was slowly added in to the mixture. The mixture was moved to a dialysis bag (MWCO = 3 KDa, Sigma, USA) to dialyze with deionized water for 24 h to remove the unloaded drug. At last, the dried solid micelles were obtained by lyophilization. The drug loading and embedding ratio were measured by the High Performance Liquid Chromatography (HPLC) (Shimadzu, Japan), and then calculated by the following formula using:

1 $${\text{Drug}}\,{\text{Loading}}\,(\% ) = {\text{weight}}\,{\text{of}}\,{\text{DEX}}\,{\text{entrapped}}/{\text{weight}}\,{\text{of}}\,{\text{nanoparticles}} \times 100\%$$

2 $${\text{Embedding ratio }}(\% ) = {\text{weight}}\,{\text{of}}\,{\text{DEX}}\,{\text{entrapped}}/{\text{weight}}\,{\text{of}}\,{\text{DEX feeding}} \times 100\%$$

### Characterization

The morphology of TKCP and TKCP@DEX was measured by transmission electron microscopy (TEM) (Bruker, Germany). The particle size distribution and zeta potential of the TKCP and TKCP@DEX were recorded using a dynamic light scattering (DLS) (Malvern, UK). The DLS was used to investigate the stability of TKCP incubated in PBS or in different concentrations of KO_2_ (0 μM, 50 μM and 100 μM) with or without ROS inhibitor (N-acetyl-l-cysteine, NAC) for 0, 4, 8, 12, 16, 20 and 24 h. The Ultraviolet–visible (UV–VIS) absorbance spectra of Cy5.5, BHQ-3 and TKCP were detected by a microplate reader (Thermo Fisher Scientific, USA).

### Fluorescent recovery of TKCP

The TKCP solution was added to different concentrations of KO_2_ (0 μM, 50 μM and 100 μM) with or without ROS inhibitor (N-acetyl-l-cysteine, NAC) and then incubated at 37 °C for 0, 1, 2, 4, 8 and 24 h, respectively. The fluorescence signals were captured by using In-vivo Multispectral Imaging Systems (Bruker, Germany).

### DEX release study

The DEX release profiles of TKCP@DEX NPs were determined by dialysis membrane method. Briefly, TKCP@DEX NPs were placed in dialysis bags and immersed in 15 mL four different buffers: (1) PBS only; (2) PBS with 50 μM KO_2_; (3) PBS with 50 μM KO_2_ and its inhibitor; and (4) PBS with 100 μM KO_2_. KO_2_ was selected as a reagent to simulate the ROS microenvironment [25]. All solutions containing 1% tween 80 were shook constantly at 37 °C. At each time point, 1 mL of aliquots was removed from the release media and 1 mL of the same buffer was supplemented. The concentration of DEX release from TKCP@DEX NPs was measured by HPLC. The procedures were performed in triplicate.

### Chondrocytes isolation and culture

Chondrocytes were isolated from the knee joints of 3-day-old C57BL6/J mice (the Animal Experimental Center of Guangxi Medical University, Nanning, China) by enzymatic digestion in aseptic conditions according to previous report [36]. Firstly, articular cartilages were digested in trypsin (Gibco, USA) for 40 min at 37 °C and then minced and digested with 2 mg/mL collagenase II for 3 h at 37 °C. Secondly, the chondrocytes were centrifuged at 1000 rpm for 5 min and then suspended in DMEM medium containing 10% fetal bovine serum (FBS, Gibco, USA), 1% penicillin and streptomycin (Solarbio, China). Then, they were transferred into a culture flask and cultured at 37 °C in a 5% CO_2_-humidified incubator. The third generation of cells was collected for further experiments.

### In vitro cytotoxicity assay

The cytotoxicity of TKCP and TKCP@DEX in chondrocytes was determined by Cell Counting Kit-8 (CCK-8, Japan). Briefly, cells were incubated in medium containing various concentrations of TKCP (0, 3, 6, 12, 25, 50, 100, 200 μg/mL) or TKCP@DEX (0, 3, 6, 12, 25, 50, 100, 200 μg/mL) for 24 and 48 h. Afterward, each well was added with 10 μL CCK-8 and incubated for 4 h in humidified incubator. The absorbance of solutions was detected at a wavelength of 450 nm by a microplate reader (Thermo Fisher Scientific, USA). CCk-8 assay was also used to assess the cell viability of MIA-induced chondrocytes after treatment with DEX, CAPP@DEX (without ROS responsive linker, TK), TKP@DEX (without chondrocyte-affinity CAP peptide, CAP), or TKCP@DEX for 24 h.

### Hemolysis test

The hemolysis ratio of TKCP at various concentrations (50, 100, 200, 400, 800 μg/mL) was performed in vitro [37]. The TKCP samples were dissolved in PBS at 37 °C. Then, 20.0 *μ*L of erythrocyte dispersion was added into the TKCP solution (1.0 mL) and the mixture was incubated for 1 h at 37 °C. After centrifuged at 2000 rpm for 10 min, the hemoglobin in supernatant was measured using a microplate reader at 415 nm. The positive and negative controls were determined by replacing the sample solution with ultrapure water and PBS, respectively. Experiments were performed for three times and the hemolysis rate (%) was calculated using following equation:

$$\left( {{\text{A}}_{{\text{s}}} - {\text{A}}_{{\text{n}}} } \right)/\left( {{\text{A}}_{{\text{p}}} - {\text{A}}_{{\text{n}}} } \right) \times 100,$$

where A_s_, A_n_, and A_p_ mean absorbencies of the sample, negative control and positive control, respectively.

### Intracellular ROS detection and bioimaging

We used monosodium iodoacetate (MIA), an inhibitor of glyceraldehyde-3-phosphate dehydrogenase activity, to induce oxidative stress injure and the pathological OA symptoms of chondrocytes [38]. Chondrocytes were seeded into 6-well or 24-well plates and divided into four groups: (1) control: chondrocytes cultured with medium only; (2) MIA 3 μM: chondrocytes induced with 3 μM MIA for 24 h; (3) MIA + inhibitor: chondrocytes pretreated with 5 mM NAC [39] (N-acetylcysteine, the antioxidant which can significantly prevent the production of ROS) for 1 h followed by addition with 3 μM MIA for 24 h; and (4) MIA 6 μM: chondrocytes induced with 6 μM MIA for 24 h. Intracellular ROS production was determined by using a fluorescent 2,7-dichlorodihydrofluorescein diacetate (DCFH-DA) kit. Chondrocytes were harvested, and incubated with DCFH-DA (10 μM) for 20 min in the dark at 37 °C. The chondrocytes were then washed three times with serum-free medium, and immediately detected by flow cytometer (BD, Biosciences, USA) [40].

In addition, cellular uptake and degradation of TKCP NPs induced by endogenous ROS were investigated. After treated with MIA, the chondrocytes were incubated with TKCP NPs for 4 h, and then they were washed with PBS and fixed with 95% ethanol for 30 min. Meanwhile, MIA (3 μM) treated cells were also incubated with CAPP and TKP NPs as controls. Then the nuclei were counterstained with DAPI. Finally, the fluorescence images were photographed using a fluorescence inversion microscope (OLYMPUS, Japan).

### MIA-induced chondrocytes and treatment

Chondrocytes were seeded into 24-well or 6-well plates and separated into five groups: (1) control: chondrocytes cultured with medium only; (2) MIA: chondrocytes induced with 3 μM MIA; (3) MIA + DEX: chondrocytes pretreated with 3 μg/mL DEX for 1 h followed by addition with 3 μM MIA for 24 h; (4) MIA + TKP@DEX: chondrocytes pretreated with TKP@DEX (an equivalent DEX dose of 3 μg/mL) for 1 h followed by addition with 3 μM MIA for 24 h; (5) MIA + CAPP@DEX: chondrocytes pretreated with CAPP@DEX (an equivalent DEX dose of 3 μg/mL) for 1 h followed by addition with 3 μM MIA for 24 h; and (6) MIA + TKCP@DEX: chondrocytes pretreated with TKCP@DEX (an equivalent DEX dose of 3 μg/mL) for 1 h followed by addition with 3 μM MIA for 24 h.

### Quantitative real-time polymerase chain reaction (RT-qPCR) analysis

The primer sequences for the OA-related genes are listed in Table 1. Total RNA was isolated using an RNA isolation kit (Tiangen Biotechnology, China). Then a reverse transcription kit (Takara, Japan) was used to reversely transcribe RNA to cDNA. Real-time PCR was conducted by a Light Cycle 96 system for 10 min at 95 °C, 15 s at 95 °C, and 60 s at 60 °C. The relative gene expression levels were calculated using the 2^−ΔΔCT^ method with β-actin as the control.

**Table 1:** Primer sequences used in RT-qPCR experiments

mRNA	Forward primer	Reverse primer
*MMP-3*	CATCCCCTGATGTCCTCGTG	GATTTGCGCCAAAAGTGCCT
*MMP-13*	TACCATCCTGCGACTCTTGC	TTCACCCACATCAGGCACTC
*IL-6*	AGCCCACCAAGAACGATAGTC	GTGAAGTAGGGAAGGCCGTG
*COL2A1*	ACACCGCTAACGTCCAGATG	TCGGTACTCGATGACGGTCT
*β-actin*	CCCATCTATGAGGGTTACGC	TTTAATGTCACGCACGATTTC

### Immunofluorescence

The expression of OA catabolic biomarkers IL-6 and MMP-13 in chondrocytes was assessed by immunofluorescence. Chondrocytes were fixed with 95% ethanol for 30 min and permeabilized with 0.1% Triton X-100 for 10 min. Samples were incubated with primary antibody as follows: IL-6 (1:200, Boster, China), and MMP-13 (1:200, Boster) at 4 °C overnight. Then the samples treated with the secondary antibodies FITC-anti-rabbit IgG (1:50, Boster) for 60 min at 37 °C and counterstained with DAPI for 5 min. Finally, the fluorescence images were photographed using a fluorescence inversion microscope (OLYMPUS, Japan).

### OA model and treatment

All animal experiments were approved by the Ethics Committee of Guangxi Medical University. A total of 60 C57BL6/J (8 weeks old, male) were obtained for this experiment. To induce OA, mice received a single IA injection of 0.05 or 0.1 mg of MIA (Sigma, USA) after anesthesia [41–43]. After induction of OA model, the mice were randomly sorted into five groups (n = 6): PBS group, IA injections of 50 μL PBS; DEX group, IA injections of 50 μL PBS with DEX (1 mg/kg); CAPP@DEX group, IA injections of 50 μL PBS with CAPP@DEX (an equivalent DEX dose of 1 mg/kg); TKP@DEX group, IA injections of 50 μL PBS with TKP@DEX (an equivalent DEX dose of 1 mg/kg) and TKCP@DEX group (an equivalent DEX dose of 1 mg/kg). IA injections were performed twice a week. The mice in these groups were sacrificed for further analysis at 2 and 4 weeks after therapy.

### In vivo NIR bioimaging

For in vivo bioimaging, mice were anesthetized by isoflurane. Each group of OA mice (n = 3) was IA injected with 50 μL of 400 μg/mL TKCP, CAPP or TKP, and the normal mice (n = 3) were also IA injected with TKCP as control. The images were captured by an In-vivo Multispectral Imaging Systems (Bruker, Germany) at 0, 1, 2, 4, 7 and 14 days.

In addition, the fluorescence intensity of the TKCP was also investigated. The mice were randomly sorted into three groups (n = 3): 0.05 mg MIA + inhibitor group, IA injection of 0.05 mg MIA concomitant with 5 mM NAC; 0.05 mg MIA group, IA injection of 0.05 mg MIA; 0.1 mg MIA group, IA injection of 0.1 mg MIA [44, 45]. Finally, the mice were sacrificed and the macroscopic evaluations of joints were performed.

### Macroscopic observation

After 2 weeks or 4 weeks of treatment, the knee joints of mice were harvested for macroscopic evaluation according to the macroscopic scoring system (scale of 0–4) by three independent observers [46].

### Histological analysis

The joints were fixed in 4% paraformaldehyde and subsequently decalcified with a 14% ethylenediaminetetraacetic acid (EDTA) solution for ten days. Next the joints were embedded in paraffin and cut into 5 μm thick slices by sharp blade. HE (Solarbio, China) and safranin O-fast green (Solarbio, China) staining were performed for histomorphological analysis. The severities of OA were graded by three independent observers by using the Osteoarthritis Research Society International (OARSI) score (scale of 0–24) [47]. Furthermore, Immunohistochemical staining for MMP13 (1:200, Boster) was performed to evaluate the anti-inflammatory effects of the probes.

### Statistical analysis

All data are presented as the mean ± SD, and p < 0.05 was considered statistically significant. The significant differences among groups were evaluated by one-way ANOVA. Statistical analyses were conducted using SPSS statistics (SPSS 19.0).

## Results

### Synthesis and fabrication of nanoreactors

**Fig. 1:**
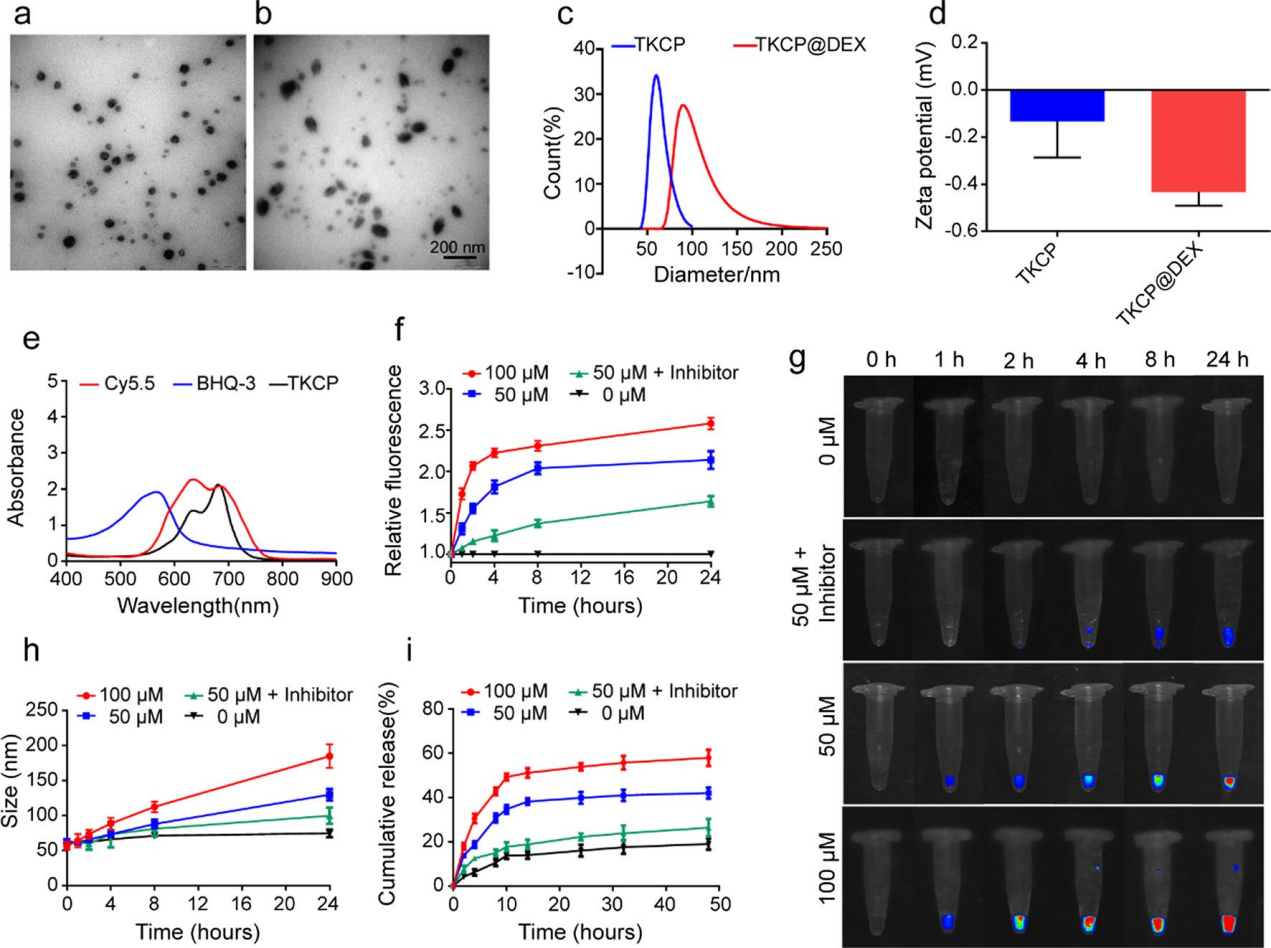
Characterization of the functionalized TKCP and TKCP@DEX. TEM images of TKCP (**a**) and TKCP@DEX (**b**) NPs. Scale bar: 200 nm. **c** DLS characterization of TKCP and TKCP@DEX NPs. **d** The zeta potential of TKCP and TKCP@DEX NPs. **e** UV–vis absorption spectra of Cy5.5, BHQ-3 and TKCP NPs. Fluorescence intensity (**f**) and Relative fluorescence intensity (**g**) of TKCP in different concentrations of KO_2_ (0 μM, 50 μM and 100 μM) or ROS inhibitor (NAC) for different times. Ex/Em of Cy5.5: 675/695 nm. **h** Size change of the TKCP NPs incubated in pH 7.4 PBS buffer containing KO_2_ (0, 50 and 100 μM) or with ROS inhibitor (NAC) for different times. **i** Cumulative DEX release from the TKCP@DEX NPs after incubation in PBS containing different concentrations of KO_2_ (0 μM, 50 μM and 100 μM) or with ROS inhibitor (NAC) for different times at 37 °C. (n = 3, mean ± SD)

The successful synthesis of ROS responsive monomer and functional moiety were demonstrated by ^1^HNMR spectra (Additional file 1: Figs. S1–S9). The transmission electron micrograph (TEM) showed that the amphiphilic polymer TKCP and TKCP@DEX could self-assemble into homogeneously spherical nanoparticles in aqueous solution (Fig. 1a and b). Furthermore, dynamic light scattering (DLS) analysis revealed that the average diameter of TKCP NPs was ~ 60 nm (Fig. 1c). The zeta potential of TKCP NPs was close to neutral charges at − 0.13 mV (Fig. 1d). After loading with DEX, the mean diameter of TKCP@DEX NPs increased up to ~ 90 nm (Fig. 1b and c) and the zeta potential changed to − 0.43 mV (Fig. 1d). The UV–vis spectrum also confirmed the successful construction of TKCP. As shown in Fig. 1e, characteristic peaks of Cy5.5 in Cy5.5-CAP-PEG-TK (TKCP) (red line) and BHQ-3 (blue line) were demonstrated. There is a red shift induced by BHQ-3 in TKCP, which is synthesized by Cy5.5-CAP-PEG-TK (TKCP) and BHQ-3 through acyl chloride. To confirm the ROS-responsive fluorescence activation properties, different concentrations of KO_2_ were added to the TKCP NPs and their fluorescence intensity was monitored. In the absence of KO_2_, the fluorescence of TKCP was extremely weak over time due to the short distance between Cy5.5 and BHQ-3 (Fig. 1f and g). However, when KO_2_ (50 μM) was added, the TKCP showed gradual recovering of NIR fluorescence over incubation time due to that the ROS cleaved the thioketal of TKCP and increased the distance between Cy5.5 and BHQ-3. The recovery of fluorescence intensity was effectively accelerated with the increasing concentration of KO_2_ from 50 to 100 μM, but it was suppressed when an ROS inhibitor NAC was added together with the KO_2_. The TKCP NPs had good stability in PBS, showing no appreciable change in size distribution. On the contrary, the size rapidly increased under different concentration of KO_2_ (50 μM and 100 μM) as the inducer of ROS, suggesting that the disassembly of TKCP NPs was triggered in response to oxidative milieu (Fig. 1h).

### In vitro drug release at ROS-simulated levels

As confirmed by the results of in vitro fluorescent recovery study (Fig. 1g), the high level of ROS in OA intracellular could break thioketal linkers and also trigger the release of DEX. The drug loading and embedding ratio were 13% and 30% for the TKCP@DEX NPs, respectively. The in vitro drug release showed that only 19% drug was released in the absence of KO_2_ (ROS inducer) after incubation for 48 h (Fig. 1i). The drug release reached up to 42% and 58% after treatment with 50 μM and 100 μM KO_2_ for 48 h, respectively, indicating obviously accelerated DEX release from the TKCP@DEX NPs by KO_2_ in a dose-dependent manner. The drug release could be inhibited by the ROS inhibitor NAC (only 26% of DEX release), because NAC could significantly prevent the production of ROS and the degradation of thioketal linkers. All these results confirmed that DEX released slowly in PBS, showing no notable change without KO_2_. On the contrary, DEX rapidly increased in the presence of KO_2_, suggesting that the disassembly of TKCP NPs was triggered by ROS according to the concentration of KO_2_.

### Cytotoxicity analysis

**Fig. 2:**
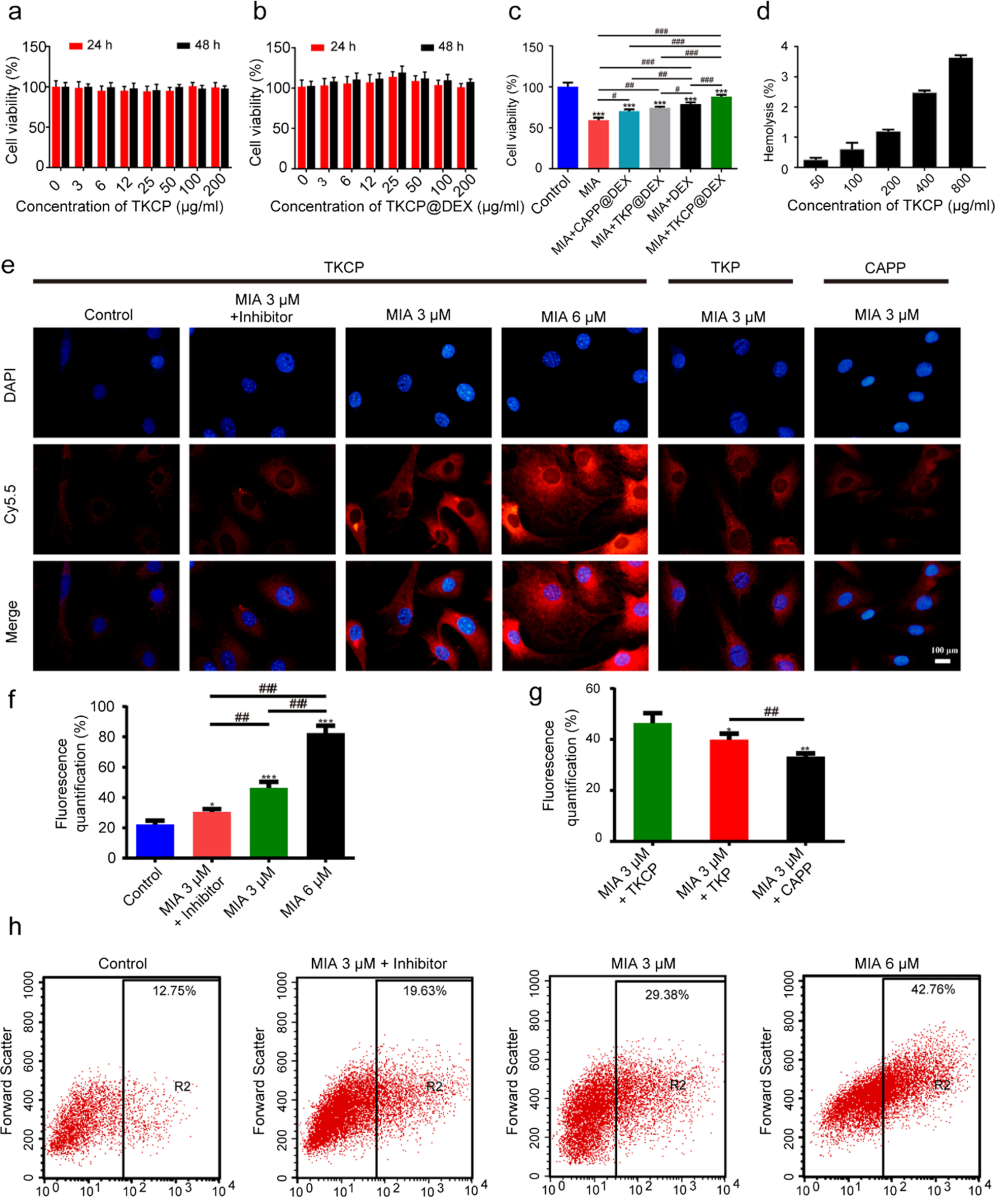
In vitro cellular evaluation. Cell cytotoxicity of TKCP against chondrocytes (**a**) and the effect of TKCP@DEX on chondrocytes (**b**) determined by the CCK-8 assay after incubating for 24 or 48 h. **c** Cell viability of MIA-induced chondrocytes pretreated with culture medium only, DEX, CAPP@DEX, TKP@DEX or TKCP@DEX for 24 h. **d** The hemolysis ratio of TKCP@DEX at different concentrations. **e** The cellular uptake of TKCP, TKP or CAPP NPs in normal chondrocytes, MIA-induced OA chondrocytes and in the presence of ROS inhibitor (NAC) for 24 h. The nuclei were counterstained with DAPI (blue). **f** Quantification of fluorescence after incubation with TKCP for different concentrations of MIA (3 μM and 6 μM) or with ROS inhibitor (NAC) for 24 h. **g** Quantification of fluorescence after incubation with TKCP, TKP or CAPP NPs in the presence of MIA (3 μM) for 24 h. Flow cytometry analysis (**h**) the production of intracellular ROS in chondrocytes at different concentrations of MIA (3 μM and 6 μM) or with ROS inhibitor (NAC) after incubation for 24 h. Scale bars: 100 µm. (n = 3; mean ± SD; *, # indicate p < 0.05; **, ## indicate p < 0.01; ***, ### indicate p < 0.001.)

The cytotoxicity of TKCP and TKCP@DEX NPs on chondrocytes was evaluated by CCK-8 assay. As show in Fig. 2a, no toxicity was observed with TKCP at the range of 0 to 200 μg/mL after 24 h and 48 h incubation. After DEX loading, 25 μg/mL TKCP@DEX (equivalent concentration of DEX was 3 μg/mL) promoted cell growth compared with untreated chondrocytes (Fig. 2b). Thus, 25 μg/mL TKCP@DEX was selected for further study.

The effect of TKCP@DEX on the cell viability of MIA (ROS inducer)-induced chondrocytes was investigated by CCK-8 analysis. As shown in Fig. 2c, the cell viability of the chondrocytes treated with MIA decreased 40.7% compared with the normal cells. However, after treating with DEX, CAPP@DEX, TKP@DEX and TKCP@DEX for 24 h, the cell viability was increased to 70.27%, 74.14%, 78.78% and 87.97% compared with the MIA group, respectively, suggesting potential function of protecting chondrocytes from catabolic activity of inflammatory factor and promoting chondrocytes proliferation.

### Hemolysis test

The biocompatibility of TKCP was measured by the hemolysis test. As shown in Fig. 2d, the TKCP at various concentrations induced lower than 4.0% hemolysis rate by contacting erythrocytes at 37 °C for 1 h. These results indicated that TKCP NPs were considered as excellent biocompatible and nonhemolytic materials, showing a great potential of biomaterials for clinical applications.

### Cell uptake and in situ fluorescent release

To investigate the chondrocytes targeting property of TKCP NPs, cellular uptake of TKCP and TKP (without chondrocyte-affinity CAP peptide) NPs were compared. Treatment with 3 μM and 6 μM MIA caused a 1.1-fold and 2.7-fold increase of red fluorescence (46.40% and 82.51%) in the cellular uptake compared with untreated cells (22.19%), respectively (Fig. 2e and f), which revealed that thioketal linkages exhibited efficient ROS-dependent degradation. The fluorescence intensity of TKCP group was observed to be much stronger than that of TKP group after treatment with 3 μM MIA (Fig. 2e and g). However, the fluorescence intensity declined to 30.55% after treating with NAC, which was similar to that of normal cell environment due to the elimination of ROS (Fig. 2e and f). These results indicated that ROS could trigger the cleavage of thioketal linkages to release Cy5.5 from TKCP according to the level of ROS at simulated pathological conditions, and the targeting effect of CAP moieties in TKCP NPs could facilitate high affinity to ECM-rich chondrocytes compared those with non-targeting modality. But in normal condition, Cy5.5 was hardly released and activated. The flow cytometer was used to monitor the ROS production in chondrocytes (Fig. 2h). At OA microenvironment induced by MIA (the concentration was 3 μM), an elevated level of ROS (29.38%) was produced compared with normal cells (12.75%). And the ROS was up to 42.76% at the concentration of 6 μM MIA, indicating a significant increase of ROS production dependent on the concentration of MIA. Furthermore, the high level of ROS could decrease down to 19.63% after treated with NAC. Thus, TKCP may potentiate smart real-time monitoring of ROS levels.

### In vitro anti-inflammatory activity

**Fig. 3:**
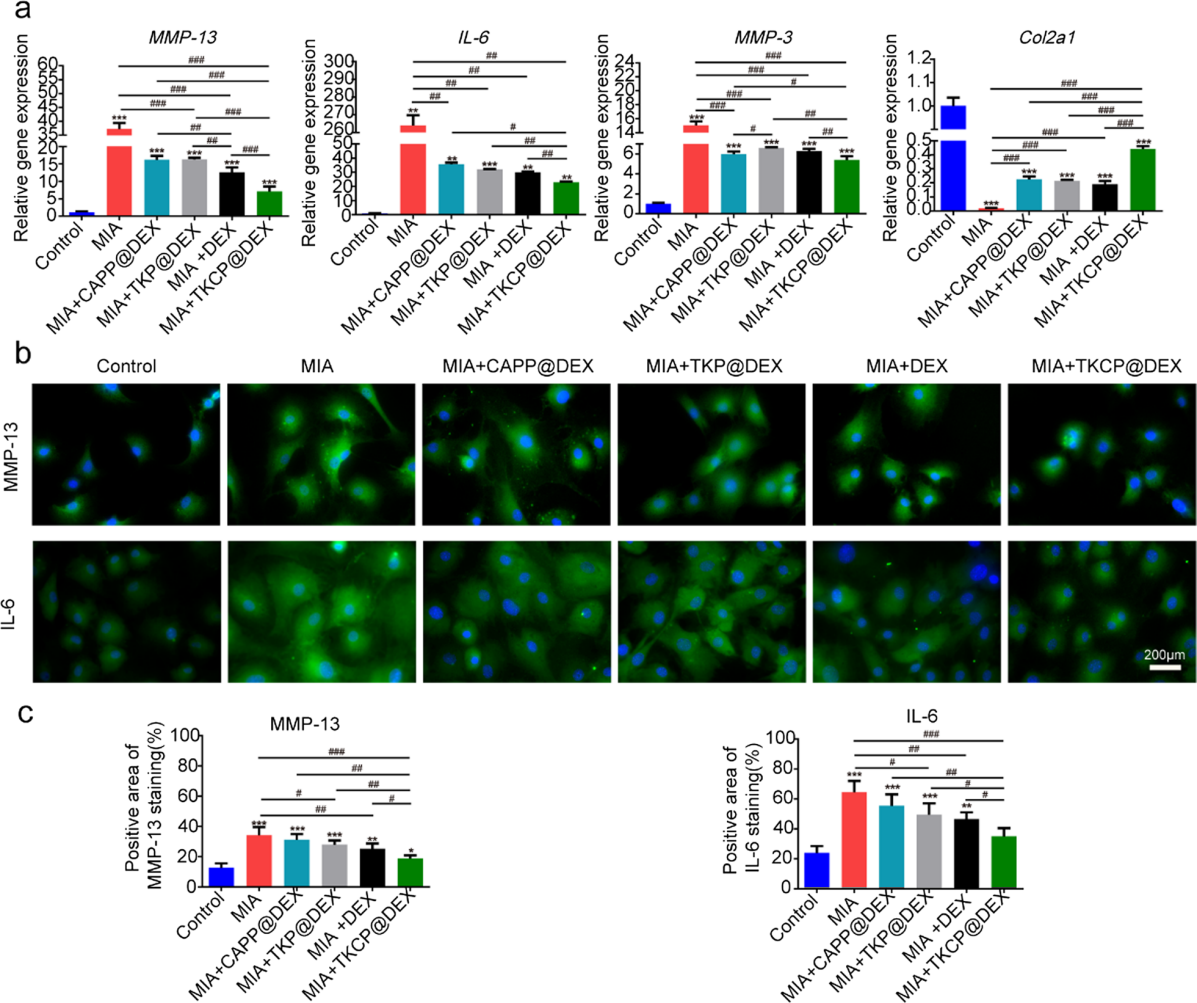
Inhibiting the action of proinflammatory factors induced by MIA, as well as protecting the chondrocytes after incubation with DEX, TKP@DEX, CAPP@DEX or TKCP@DEX. **a** Relative mRNA levels of *MMP-13*, *Il-6*, *MMP-3* and *Col2a1* on MIA-stimulated chondrocytes after incubation with DEX, CAPP@DEX, TKP@DEX or TKCP@DEX. **b** Immunofluorescence images of MIA-stimulated chondrocytes after incubation with DEX, CAPP@DEX, TKP@DEX or TKCP@DEX. **c** Quantifying the level of MMP-13and IL-6 after treating with DEX, CAPP@DEX, TKP@DEX or TKCP@DEX for 24 h. Scale bar: 200 μm. (n = 3; mean ± SD; *,# indicate p < 0.05; **, ## indicate p < 0.01, ***; ### indicate p < 0.001.)

We then evaluated the efficacy of treatment groups to induce crucial OA catabolic biomarkers (*MMP-13, IL-6, and MMP-3*) and chondrogenic markers genes (*Col2a1*) by qRT-PCR (Fig. 3a). It showed that the *MMP-13, IL-6 and MMP-3* expression of TKCP@DEX group was significantly decreased than that of the MIA-treated group, down to 80.85%, 91.30% and 64.19%, respectively. Compared with DEX, there were 43.47%, 23.37% and 14.19% decreases of MMP13, IL-6 and MMP-3 expression in TKCP@DEX group respectively, indicating its pronounced anti-inflammatory effect. TKCP@DEX group also significantly up-regulated the expression of *Col2a1,* one key component of the cartilage matrix compared with other groups after 24 h of culture (Fig. 3a), suggesting that TKCP@DEX NPs inhibited the degradation of collagen II and protected chondrocytes under the pathological conditions of OA. The expression of OA biomarkers by immunofluorescent staining further confirmed the PCR results (Fig. 3b and c). The results showed the expressions of MMP-13 and IL-6 were both decreased in the TKCP@DEX group, almost 25–45% lower than other groups (MIA, DEX, CAPP@DEX and TKP@DEX groups).

### In vivo ROS-responsive activity

**Fig. 4:**
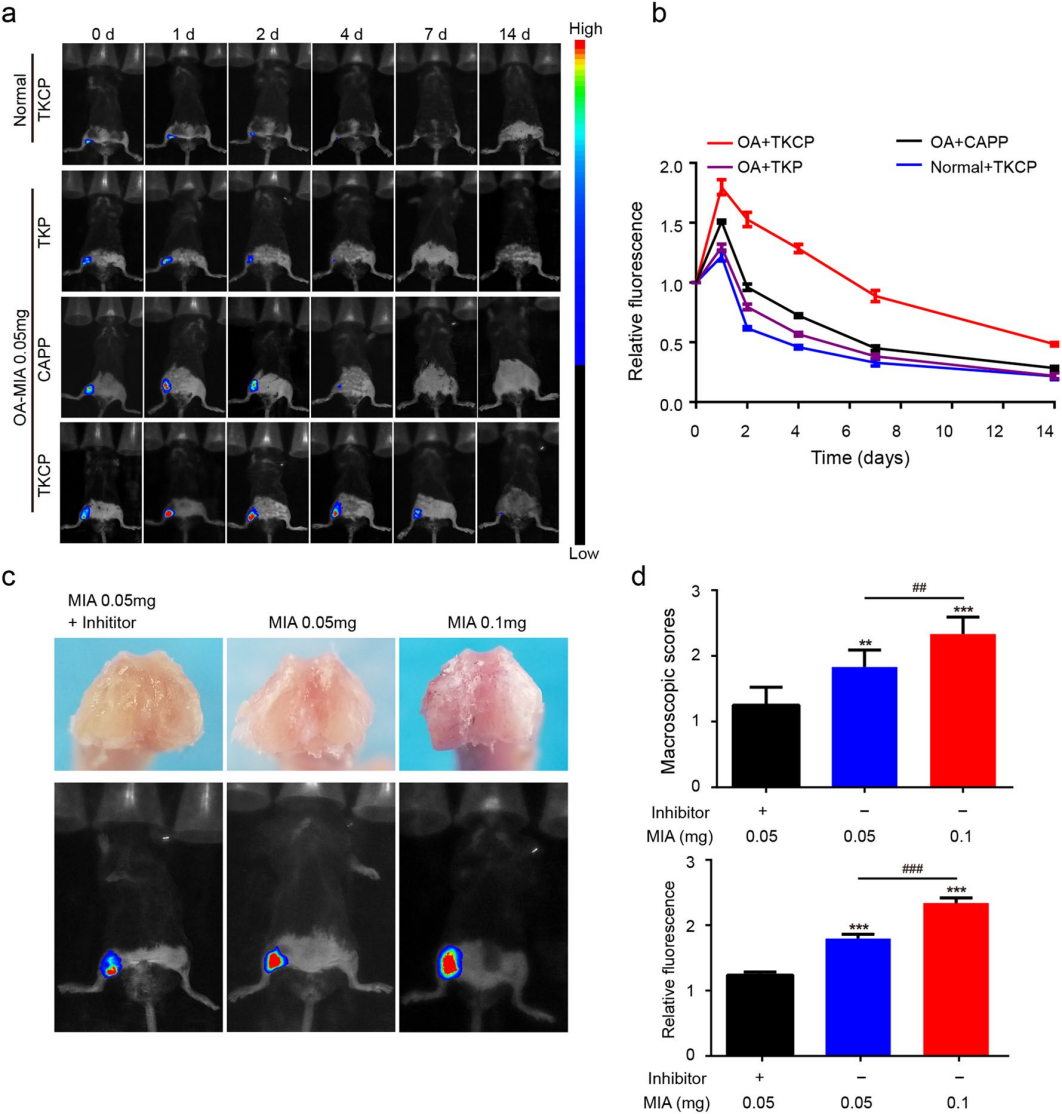
In vivo fluorescent image after IA injection of CAPP, TKP or TKCP probes to detect the level of ROS induced by MIA in C57BL6/J knees. **a** The up-regulated ROS activity in the OA joints was reflected by fluorescent signal and measured by an in vivo imaging system at selected time points after IA-injection. Ex: 630 nm, Em: 700 nm. **b** The relative fluorescence intensity of TKCP, CAPP or TKP probes at different treatment times. **c** Representative photographs showed the macroscopic appearance of the cartilage from the femoral condyles and in vivo NIR bioimaging indicated the level of ROS via the fluorescent intension according to the development of OA. **d** Analysis of the macroscopic score and relative fluorescent intensity to evaluate the severity of OA. (n = 3; ean ± SD; *, # indicate p < 0.05, **; ## indicate p < 0.01; ***, ### indicate p < 0.001.)

To detect the ROS activity at the early stage of OA, we established the OA model by IA injection of MIA (ROS inducer). In the normal group (healthy joints) treatment, IA injection of TKCP probes resulted in weak fluorescent signal in the normal group (healthy joints) and the relative fluorescence intensity was 1.20 at 1 day (Fig. 4a and b). By contrast, a strong fluorescent signal was detected in the OA joints, indicating significantly up-regulated ROS activity in the OA joints compared to healthy joints. The relative fluorescence intensity of TKCP group in OA sites reached peak intensity at 1 day, and still maintained obvious fluorescence at 14 days. Moreover, the relative fluorescence intensity of TKCP NPs treated mice was nearly ~ 1.34-fold higher than that of CAPP NPs, and ~ 0.95-fold higher than TKP NPs (Fig. 4a and b) at 7 days, indicating that the decoration of CAPP endowed the potential enrichment effect of TKCP NPs and thioketal linkage could be effectively cut off by ROS in cartilage. Thus, TKCP NPs exhibited cartilage targeting property and prolonged retention time in joints, which was suitable for effective bioimaging to detect the development of OA in vivo.

Compared with the OA mice (0.05 mg MIA) injected with the TKCP probes, those injected with the TKCP + ROS inhibitor group produced weaker fluorescence signal (Fig. 4c and d). In addition, when mice injected with 0.1 mg MIA, a higher ROS circumstance was induced, leading to more serious joint damage (Fig. 4c and d). And the relative fluorescence intensities could be increased at higher ROS circumstance (IA with 0.1 mg MIA), increasing up to 30% higher than that of IA with 0.05 mg MIA after treated with TKCP probes. This result indicated that the intensity of fluorescent signals of TKCP probe could respond to ROS related OA severity, favorable for precise disease classification.

### In vivo treatment effect

**Fig. 5:**
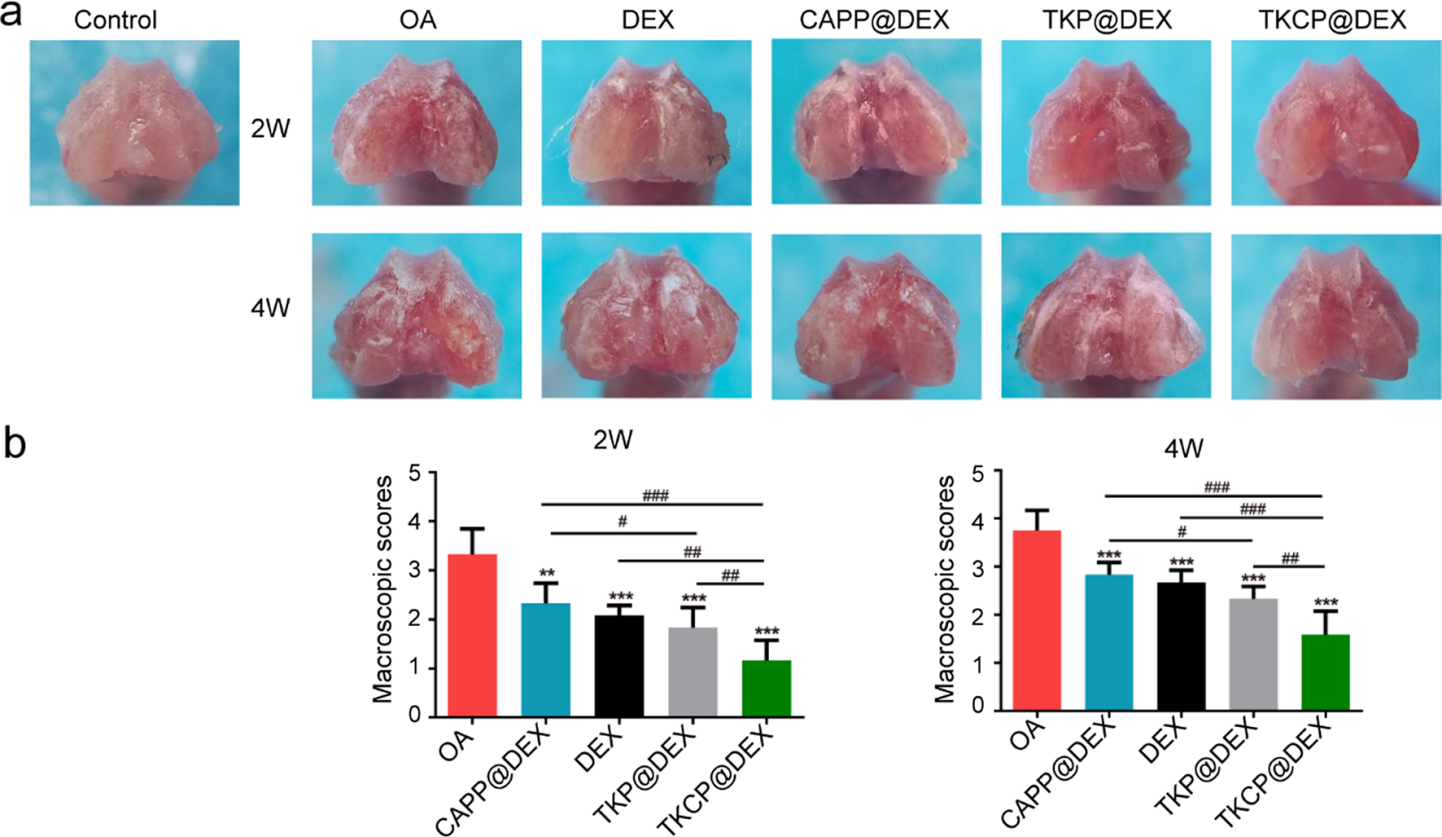
Macroscopic appearance and scoring of cartilage femoral condyles after treatment for 2 and 4 weeks. **a** The macroscopic observation and **b** the macroscopic scores of cartilages after IA-injection with PBS, DEX, CAPP@DEX, TKP@DEX or TKCP@DEX (n = 6; mean ± SD)

We further carried out in vivo experiments to evaluate the OA therapeutic efficacy of TKCP@DEX. The mice were divided into six groups, including PBS, OA, DEX, CAPP@DEX, TKP@DEX and TKCP@DEX. In the PBS-treated group, general characteristic OA features, such as surface irregular and large cartilage erosion, were observed. The macroscopic score of OA group was significantly higher than that of control group over time, which indicated mild or moderate cartilage destruction based on pathological alteration of joint morphology from 2 to 4 weeks (Fig. 5a). After treatment with DEX, there was a 37.50% and 28.89% reduction in the joint score in 2 weeks and 4 weeks, respectively, as compared to that of treatment with PBS alone. Meanwhile, both CAPP@DEX and TKP@DEX treatment significantly decreased the severity of OA. Furthermore, there was a 65% and 57.78% reduction in TKCP@DEX group after treatment for 2 and 4 weeks, respectively, compared to that OA group, which showed its effective therapeutic repair effect of OA (Fig. 5b).

**Fig. 6:**
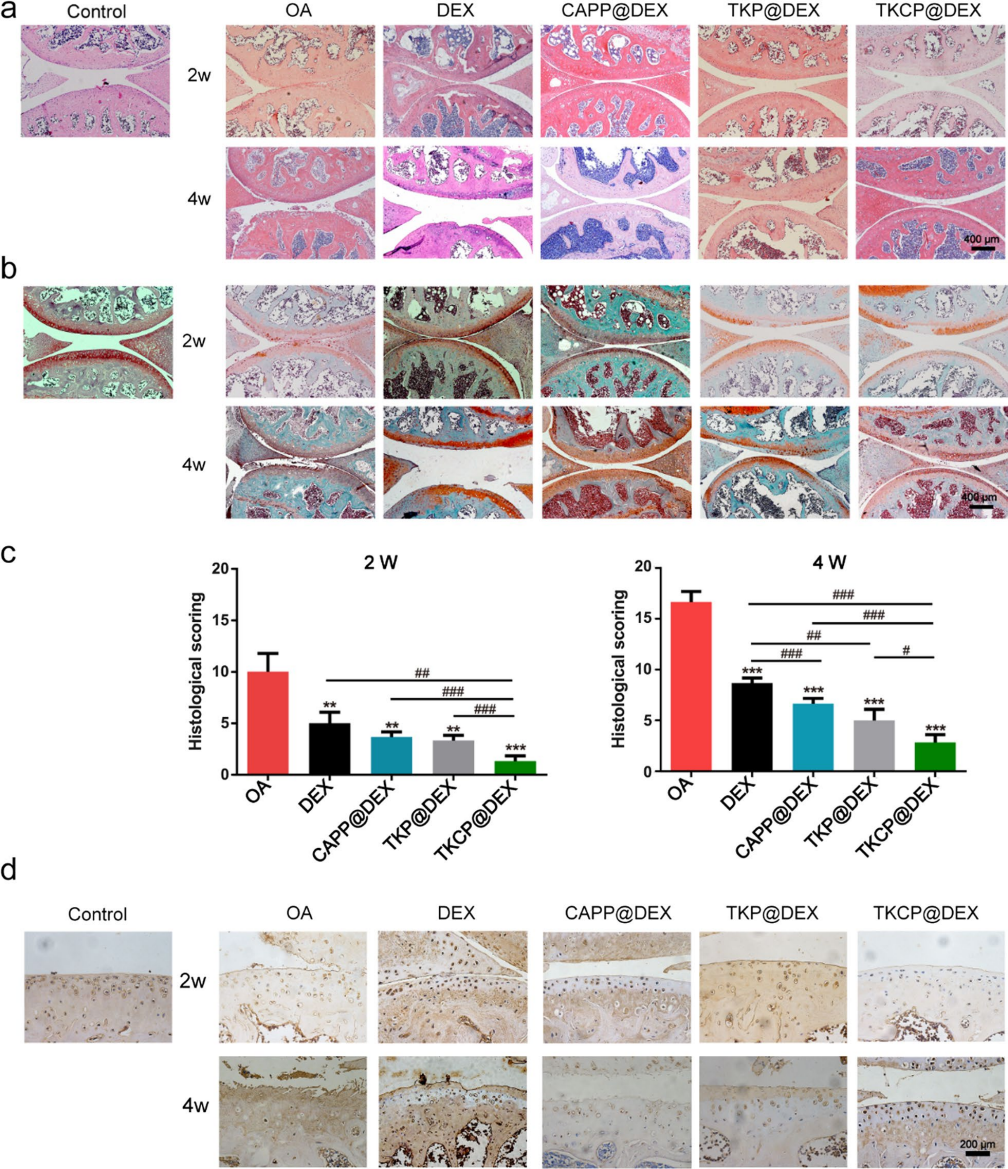
Histological evaluations of cartilage femoral condyles after IA injection of probes for 2 weeks and 4 weeks. H&E (**a**) and safranin-O/fast green (**b**) staining of cartilage sections. Scale bar: 400 μm. **c** Histological scoring of articular cartilage. **d** Immunohistochemical staining of MMP-13 was evaluated on cartilage sections after IA-injection with PBS, DEX, CAPP@DEX, TKP@DEX or TKCP@DEX. Scale bar: 200 μm. (n = 6; mean ± SD; *, # indicate p < 0.05, **; ## indicate p < 0.01; ***, ### indicate p < 0.001.)

The HE and safranin O/fast green staining were used to evaluate the therapy efficacy of TKCP@DEX in vivo (Fig. 6a and b). In the OA group, OA characteristics, including cartilage damage, fibrillated lesions and loss of aggrecan worsened over time, were showed in the progression of OA. In contrast, TKCP@DEX treatment resulted in a significant improvement in matrix arrangement, tide line maintenance or cartilage lesion, and the OARSI score exhibited improvement, with a reduce of 86.7% and 83% compared with OA group at 2 and 4 weeks, respectively (Fig. 6c). The scores for the TKCP@DEX group were markedly lower than that of other treatment groups, exhibiting positive effects on the restoration of cartilage matrix expression.

We also assessed the expression of MMP-13 in OA cartilage by immunohistochemistrical analysis, which is a degradation product of type II collagen and corresponds to articular cartilage damage in the early stages of OA. The positive staining of MMP-13 could hardly be detected in the normal cartilage. In contrast, it showed high expression in the superficial and middle zones of the cartilage in OA group. Following treatment with DEX, CAPP@DEX, TKP@DEX or TKCP@DEX, osteoarthritic changes of MMP-13 over-expression was reduced (Fig. 6d). These results indicated that TKCP@DEX significantly slowed the progression of early OA and prevented the severe damage to articular cartilage in the OA.

## Discussion

The abnormal over-production of endogenous ROS within cartilage tissue is a key hallmark of OA [48], providing a disease-specific triggering mechanism for drug control and release systems. Herein, we fabricated an advance ROS responsive and cartilage targeting TKCP@DEX nanoprobe with loaded drug for imaging and effective therapy of OA, which may provide reference for clinical application.

As a promising domain, stimuli responsive theranostic nanoprobes have been widely applied in disease theranostic owing to their unique all-in-one features [49–51]. Several researchers proposed ultrasensitive ROS-responsive carriers that contained agents to release the drug to the disease site [48, 52, 53]. Compared with unresponsive delivery systems, these ROS-responsive systems remain stable in normal tissue, preventing their release to non-inflamed tissues, which suggested their specificity in inflammation site. But these ROS responsive nano systems can only control the release of drugs, they cannot visually real-time monitor the progression of disease and most of them have no therapeutic effect. In our study, TKCP is superior to unresponsive CAPP, as evidenced by fluorescent recovery studies simulated by abundant of ROS in vitro and vivo (Figs. 1f, g, 2e and 4a). The fluorescent signal is strong in the OA microenvironment, but is extremely weak in normal chondrocytes and joints, indicating that the level of fluorescence signal correlates with ROS content. TKCP@DEX not only exhibits smart drug release potential in response to high levels of ROS as demonstrated by drug release behavior test (Fig. 1i), but also can real-time track ROS activity since it emits signal of fluorescence varied with ROS, promising for monitoring and on-demand therapy of OA.

On the other hand, cartilage targeting is of importance for drug therapy. Most in situ injected particles may be quickly cleared by the joint fluid because of the inability of homing to lesion sites, leading to application limitations in OA diagnosis and treatment. Pi et al. engineered a chondrocyte-affinity peptide (CAP, DWRVIIPPRPSA) [32] by phage display technology. In our study, the nanoprobe TKCP with cartilage targeting CAP showed stronger fluorescence than TKP (without CAP), as observed in vitro cellular uptake tests (Fig. 2e) and in vivo NIR imaging (Fig. 4). Moreover, the CAP-modified TKCP@DEX prolonged the retention time of nanoparticles in the joint (Fig. 4a and b), which could improve therapeutic outcome of OA. These results indicated that cartilage targeting ensured the real-time monitoring and effective treatment of OA.

In our studies, we observed that the TKCP@DEX NPs showed stronger therapeutic efficacy than that of delivery systems without conjugation of CAP and TK. The TKCP@DEX NPs play a key role in relieving the inflammation in vitro and in vivo (Figs. 3, 5, 6). The results of macroscopic and histological analysis further evaluated that IA injection of TKCP@DEX showed the better therapeutic effect than other formulations (TKP@DEX, CAPP@DEX or free DEX) after 2 and 4 weeks of treatment (Figs. 5, 6). In addition, hemolysis test further assessed the excellent biocompatibility of the TKCP@DEX NPs platform (Fig. 2d). The above results revealed that the drug delivery system targeted to the cartilage and responded well to low concentrations of ROS, exhibiting highly sensitive imaging and effective anti-inflammatory activity for therapy of OA.

## Conclusions

In summary, we have successfully fabricated a novel cartilage-targeting and ROS-responsive delivery platform for imaging and therapy of OA, which enables real-time imaging to monitor the severity of OA and on-demand drug release at the site of abnormal ROS milieu. We expect that this theranostic system may be applied in clinic for treatment of OA.

## Supplementary Information

**Additional file 1.** All data generated or analysed during this study are included in this published article and its additional files.
