# Supplementary material for: Reactive oxygen species (ROS)-responsive nanoprobe for bioimaging and targeting therapy of osteoarthritis
Source: J Nanobiotechnology. 2021 Nov 27;19:395. doi: 10.1186/s12951-021-01136-4 (PMC8627084; doi:10.1186/s12951-021-01136-4)
Supplement: Supplementary file 1 — Additional file 1. All data generated or analysed during this study are included in this published article and its additional files. [file 12951_2021_1136_MOESM1_ESM.docx]

**Reactive Oxygen Species (ROS)-responsive nanoprobe for bioimaging and targeting therapy of osteoarthritis**

**Materials**

3-mercaptopropionic acid (98%), 1-ethyl-3-(3-dimethylaminopropyl) carbodiimide hydrochloride (EDC, 97%), N-hydroxysuccinimide (NHS, 98%), N,N-Dimethylformamide (DMF, 98%), 4-dimethylaminopyridine (DMAP),  *N*, *N*-Diisopropylethylamine (DIPEA, 98%), ethanolamine, 1,6-hexanediol (HDO), tetrahydrofuran (THF), dichloromethane (DCM), Cy5.5-NHS, BHQ3-NHS and Dexamethasone (DEX, 98%) were purchased from Sigma-Aldrich (China). NH_2_-PEG-NH_2_ with a molecular weight (MW) of 3400 Da (95%), was purchased from Xi’an Ruixi Biological Technology Co., Ltd. (Xi’an, China). Chondrocyte-affinity peptide (CAP, DWRVIIPPRPSA) was purchased from GL Biochem (Shanghai, China). Potassium superoxide (KO_2_) was provided by Alfa Aesar.

**Synthesis of thioketal containing monomer (TK).**

A mixture of anhydrous 3-mercaptopropionic acid (5.2 g, 49.1 mmol) and anhydrous acetone (5.8 g, 98.2 mmol) was saturated with dry hydrogen chloride and stirred at room temperature for 6 h. After the reaction, the flask was placed in an ice salt bath and rapidly cooled until crystallization was completed (**Figure** **S1**). Subsequently, the product was filtered and washed with hexanes and cold water for three times to obtain the crude product, which was then purified *via* HPLC and verified *via* ^1^HNRM (ADVANCE III HD600 600MHz spectrometer, Bruker, Germany). ^1^H NMR (600 MHz, DMSO-d^6^) δ:2.74 (t, 4H), 2.50 (t, 4H), 1.53 (s, 6H).

**Synthesis of the BHQ3-OH.**

The ethanolamine (4 mg) and BHQ3-NHS ester (2 mg) were dissolved in 200 *μ*L of anhydrous DMF, respectively. Then DIPEA (10 *μ*L) was added to the ethanolamine solution and shaken at room temperature for 10 min. After the reaction, the ethanolamine solution was transferred into BHQ3-NHS ester (2 mg) and was stirred at room temperature for 3 h. Finally, the reaction was terminated by adding 10% TFA to obtain the product BHQ3-OH. The synthesized product (BHQ3-OH) was purified by HPLC and verified by ^1^H NRM (**Figure** **S2**).

**Synthesis of Cy5.5-CAP-PEG-NH_2_ and Cy5.5-PEG-NH_2._**

Firstly, the targeting peptide (CAP) was labeled with Cy5.5 to prepare Cy5.5-CAP. The chondrocyte targeting peptide (CAP, 5 mg) and Cy5.5-NHS ester (2 mg) were separately dissolved in 200 *μ*L of anhydrous DMF, then 10 *μ*L of DIPEA was added, respectively, and shaken at room temperature for 10 min. Then, the solution was mixed and was stirred at room temperature in dark for 3 h. To conjugate Cy5.5-CAP to one terminal of NH_2_-PEG-NH_2_ to generate the product Cy5.5-CAP-PEG-NH_2_, the Cy5.5-CAP (36 mg), EDC (2.3 mg) and NHS (2.18 mg) were dissolved in DMF (3 mL) and stirred at room temperature for 0.5 h, then the mixture was transferred into the NH_2_-PEG-NH_2_ (100 mg) solution and stirred at room temperature for 48 h. The Cy5.5-CAP-PEG-NH_2_ was obtained after dialysis for three days and lyophilization under vacuum. The synthesis route is shown in **Figure S3**. The ^1^HNMR spectrum of Cy5.5-CAP-PEG-NH_2_ is shown in **Figure S7.**

To obtain Cy5.5-PEG-NH_2_, a mixture of Cy5.5-NHS ester (5 mg), NH_2_-PEG-NH_2_ (50 mg) were dissolved in 1 mL anhydrous DMF. Then, 10 *μ*L of DIPEA was added and the mixture was shaken at room temperature for 24 h. The synthesis route of Cy5.5-PEG-NH_2_ is shown in **Figure S4.**

**Synthesis of Cy5.5-CAP-PEG-TK and Cy5.5-PEG-TK.**

Thioketal containing monomer (50 mg, 0.4 mmol), EDC (2.8 mg), and NHS (1.96 mg) are dissolved in 1 mL of DMF and the mixture was stirred for 1 h under a nitrogen atmosphere. The reaction mixture was stirred at room temperature for 0.5 h. After the reaction, the solution was transferred into the Cy5.5-CAP-PEG-NH_2_ (50 mg) or Cy5.5-PEG-NH_2_ (50 mg) solution, and stirred at room temperature for 48 h. After dialysis for three days, it was lyophilized in vacuo to obtain the product Cy5.5-CAP-PEG-TK or Cy5.5-PEG-TK. The synthesis route is shown in **Figure S3 and Figure S4.** The ^1^HNMR spectrum of Cy5.5-CAP-PEG-TK is shown in **Figure S8.**

**Preparation of the Cy5.5-CAP-PEG-TK-BHQ3 (TKCP), Cy5.5-PEG-TK-BHQ3 (TKP) and Cy5.5-CAP-PEG-BHQ3 (CAPP).**

Synthesis of Cy5.5-CAP-PEG-TK-BHQ3 (TKCP): The solution of Cy5.5-CAP-PEG-TK (291.7 mg, 0.66 mmol), triethylamine (TEA, 2 mmol), 2, 4, 6-trichlorobenzoylchloride (169.3 mg, 0.7 mmol), and 4-dimethylaminopryidine (DMAP, 24.4 mg, 0.2 mmol) in anhydrous DMF (5 mL) was added to the above mentioned solution and the mixture was stirred at room temperature for 10 min. Next, the solution of BHQ3-OH (20 mg, 0.133 mmol) in 4 mL of anhydrous DMF was added and the reaction was stirred for 24 h at room temperature. Then, solution was added dropwise into 70 mL stirred deionized water and stirring was continued for 4 h. Next, the solution was loaded into a dialysis bag (MWCO =2 KDa) and dialyzed against deionized water for 48 h. The purified intermediate Cy5.5-CAP-PEG-TK-BHQ3 (TKCP) was lyophilized (188.3 mg, 71.2% yield) and stored at -20 °C for further use (**Figure S3**). The ^1^HNMR spectrum of Cy5.5-CAP-PEG-TK-BHQ3 is shown in **Figure S9.**

Synthesis of Cy5.5-PEG-TK-BHQ3 (TKP): The reaction bottle is connected to the exhaust gas absorption device. Cy5.5-PEG-TK (100 mg) and thionyl chloride was mixed and stirred at 58-62 °C. The temperature was maintained until the reaction was stopped in the exhaust gas absorption bottle, and the reaction was stopped and distilled under reduced pressure. Thereafter, the BHQ3-OH (20 mg, 0.133 mmol) was dissolved in dichloromethane and stirred in an ice water bath at 0-5 °C. The acid chloride chlorinated Cy5.5-PEG-TK was dissolved in dichloromethane and added dropwise to the mixed solution at 0-5 ° C for 1 h. The mixture was heated to 20 °C and stirred for 2 h. Finally, the mixture was lyophilized by dialysis to obtain the final product Cy5.5-PEG-TK-BHQ3 (TKP) (**Figure S4**). The ^1^HNMR spectrum is shown in **Figure S10.**

Synthesis of Cy5.5-CAP-PEG-BHQ3 (CAPP): The solution of Cy5.5-CAP-PEG-NH_2_ (841 mg, 0.4 mmol), EDCI (153 mg, 0.8 mmol), N-succinimidyl 3-maleimidopropionate (212 mg, 0.8 mmol) was added to anhydrous DMF (3 mL) and the mixture was stirred at room temperature for 2 h. Next, a certain of 3-mercaptopropionic acid in 2 mL of anhydrous DMF was added and the reaction was stirred for 24 h at room temperature. Then, the solution was loaded in a dialysis bag (MWCO =2 KDa) and dialyzed against deionized water for 48 h. The Cy5.5-CAP-PEG-BHQ3 was synthesized according to the protocol of TKCP. Briefly, the above purified intermediate (190 mg, 0.08 mmol), triethylamine (TEA, 16.3 mg, 0.16 mmol ), 2, 4, 6-trichlorobenzoylchloride (20 mg, 0.08 mmol), and 4-dimethylaminopryidine in anhydrous DMF (5 mL) was added to the solution of BHQ3-OH (50 mg, 0.084 mmol) in 4 mL of anhydrous DMF, and the reaction was stirred for 24 h at room temperature. Then, solution was added dropwise into 20 mL stirred deionized water and stirring was continued for 4 h. Next, the solution was loaded into a dialysis bag (MWCO =2 kDa) and dialyzed against deionized water for 48 h. The purified intermediate Cy5.5-CAP-PEG-BHQ3 (CAPP) was lyophilized (188.3 mg, 71.2% yield) and stored at -20 °C for further use (**Figure S5**). The ^1^HNMR spectrum of Cy5.5-CAP-PEG-BHQ3 is shown in **Figure S10.**

**Synthesis of** [**Cy5.5-CAP-PEG-TK-BHQ3@DEX**](mailto:Cy5.5-CAP-PEG-TK-BHQ3@DEX) **(TKCP @DEX),** [**Cy5.5-PEG-TK-BHQ3@DEX**](mailto:Cy5.5-PEG-TK-BHQ3@DEX) **(TKP@DEX) and** [**Cy5.5-CAP-PEG-BHQ3@DEX**](mailto:Cy5.5-CAP-PEG-BHQ3@DEX) **(CAPP@DEX)**

The DEX (10 mg) and TKC-PEG (20 mg) were dissolved in tetrahydrofuran (500 μL), then mixed two and added with water (10 mL) to sonicate for 20 minutes and stir for 0.5 h. The mixture was dialyzed to remove the unloaded drug, and lyophilized to obtain TKCP@DEX. The TKP@DEX (without CAP) and CAPP@DEX (without TK) were synthesized according to above report.

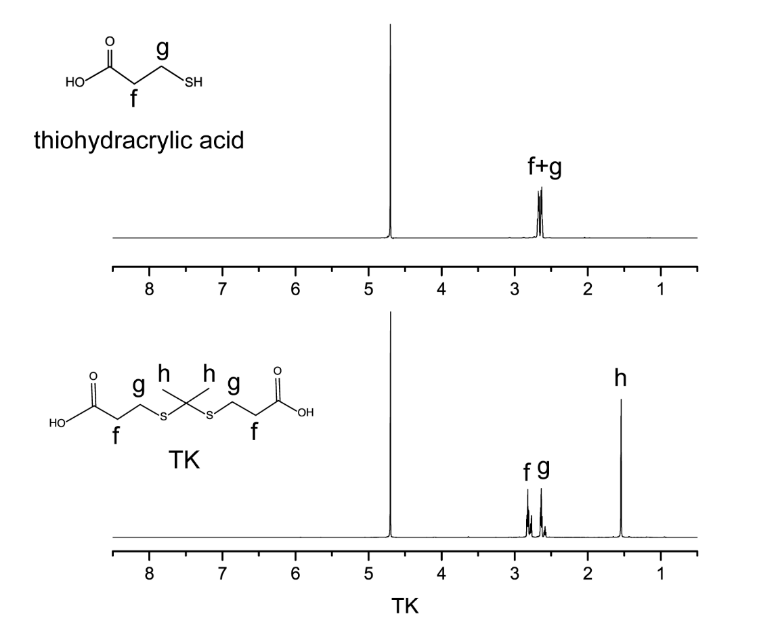


**Figure S1**. Synthesis route of TK and ^1^HNRM spectrum of TK in D_2_O.

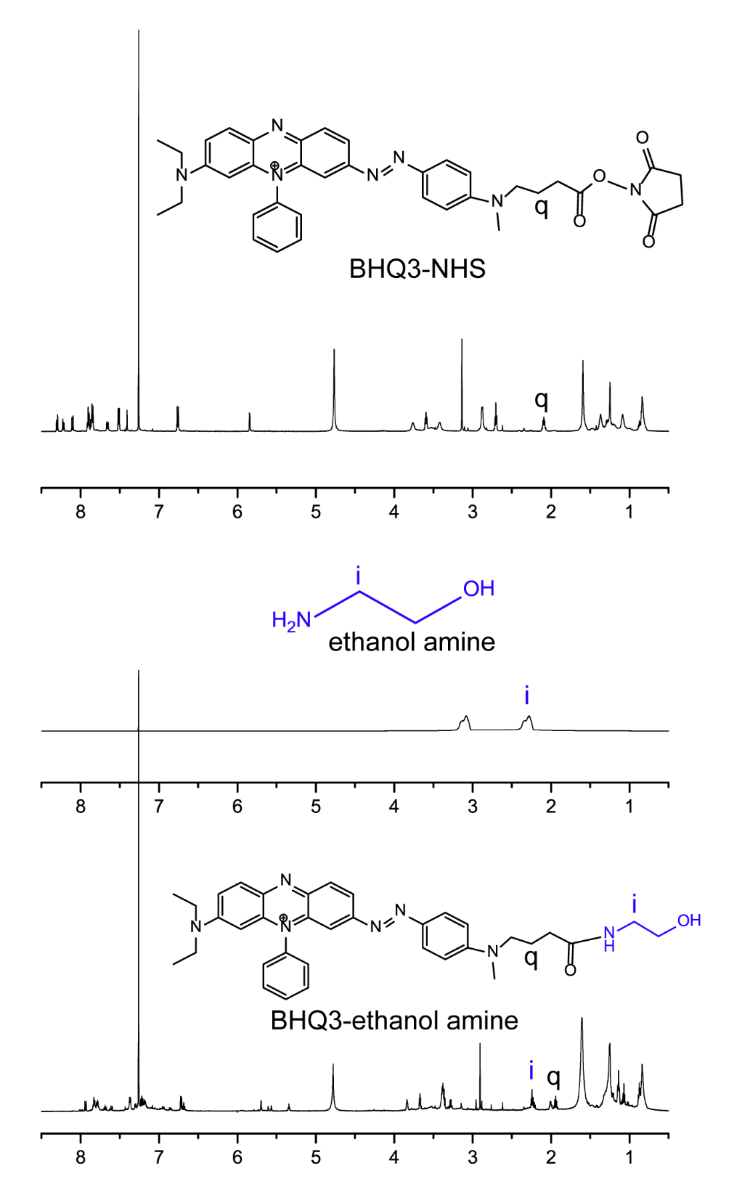


**Figure S2**. Synthetic pathway toward 3-BHQ-ethanol amine and ^1^HNMR spectrum of BHQ3-ethanol amine in CDCl_3._

**Figure S3**. Synthetic pathway toward Cy5.5-CAP-PEG-TK-BHQ3 (TKCP).

**Figure S4**. Synthetic pathway toward Cy5.5-PEG-TK-BHQ3 (TKP).

**Figure S5**. Synthetic pathway toward Cy5.5-CAP-PEG-BHQ3 (CAPP).


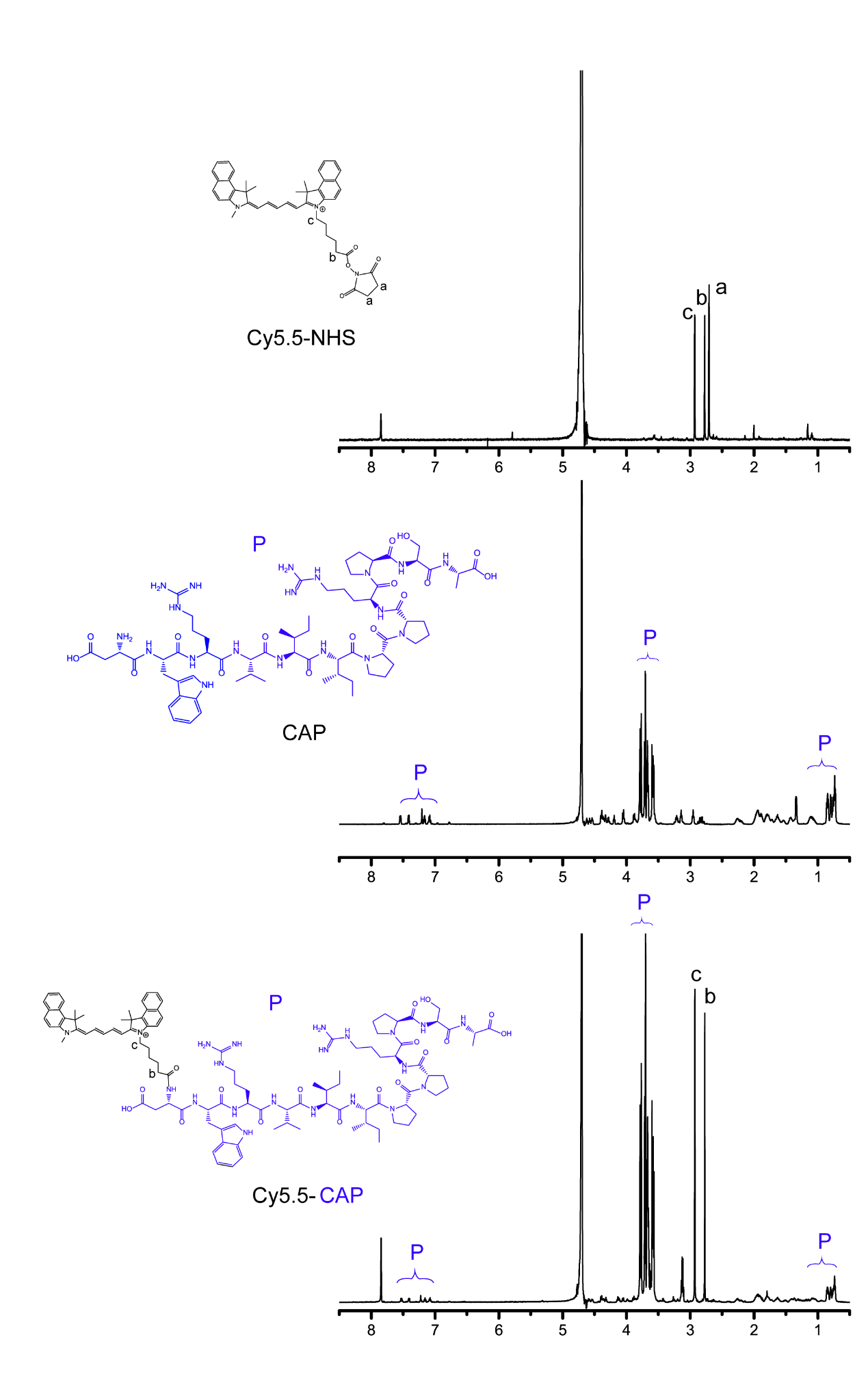


**Figure S6.** ^1^HNMR spectrum of Cy5.5-CAP in D_2_O.


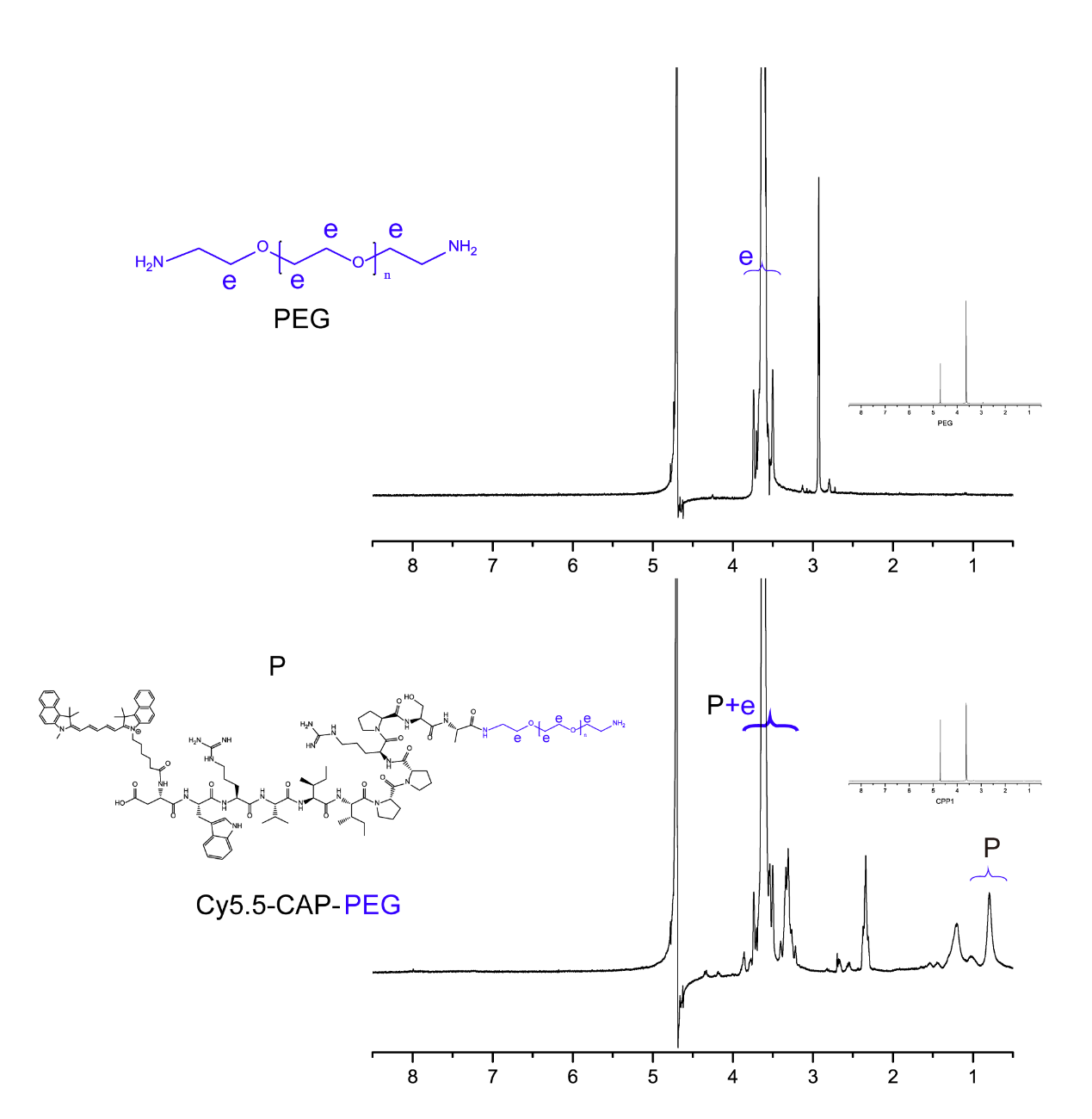


**Figure S7.** ^1^HNMR spectrum of Cy5.5-CAP-PEG in D_2_O.


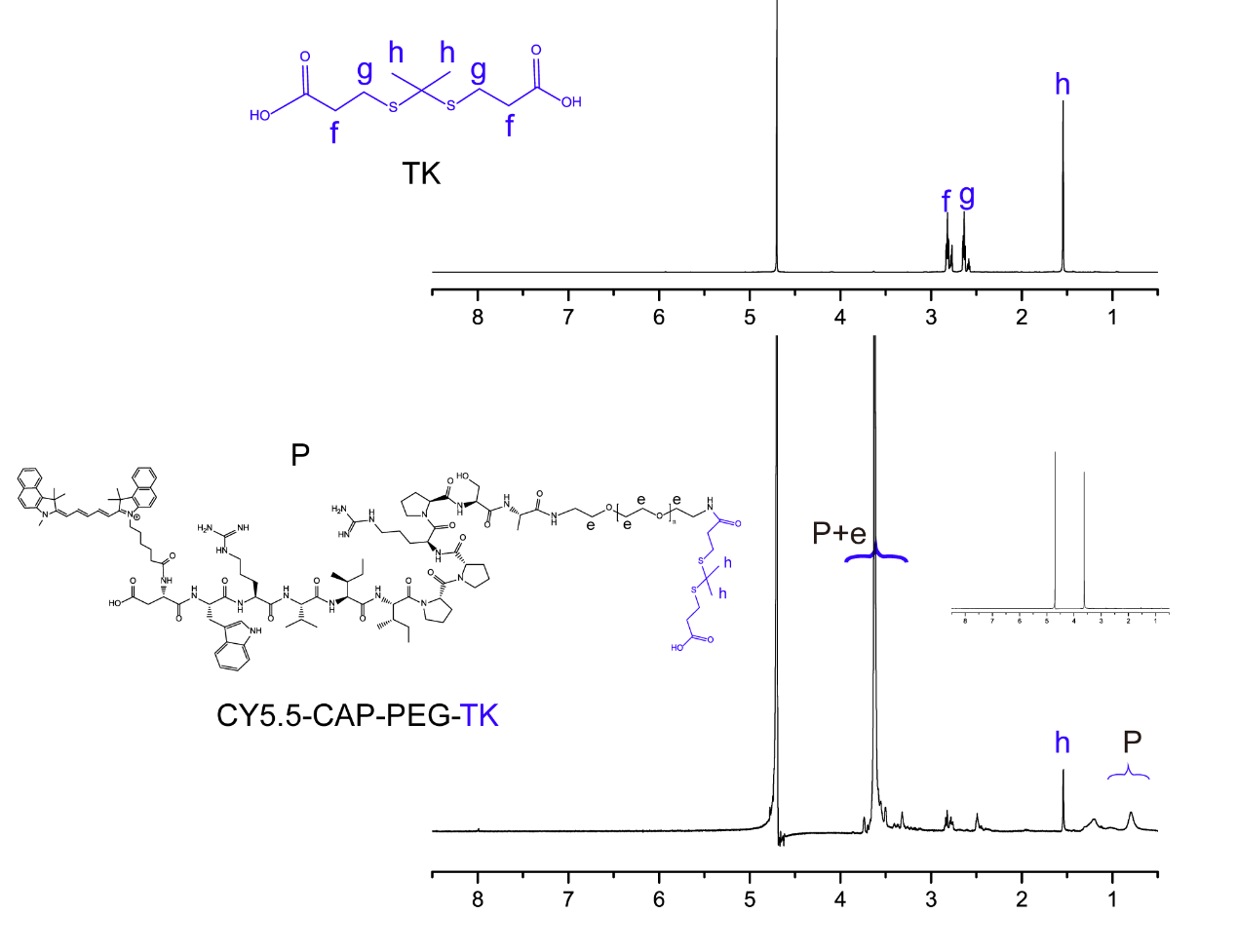


**Figure S8.** ^1^HNMR spectrum of Cy 5.5-CAP-PEG-TK in D_2_O.


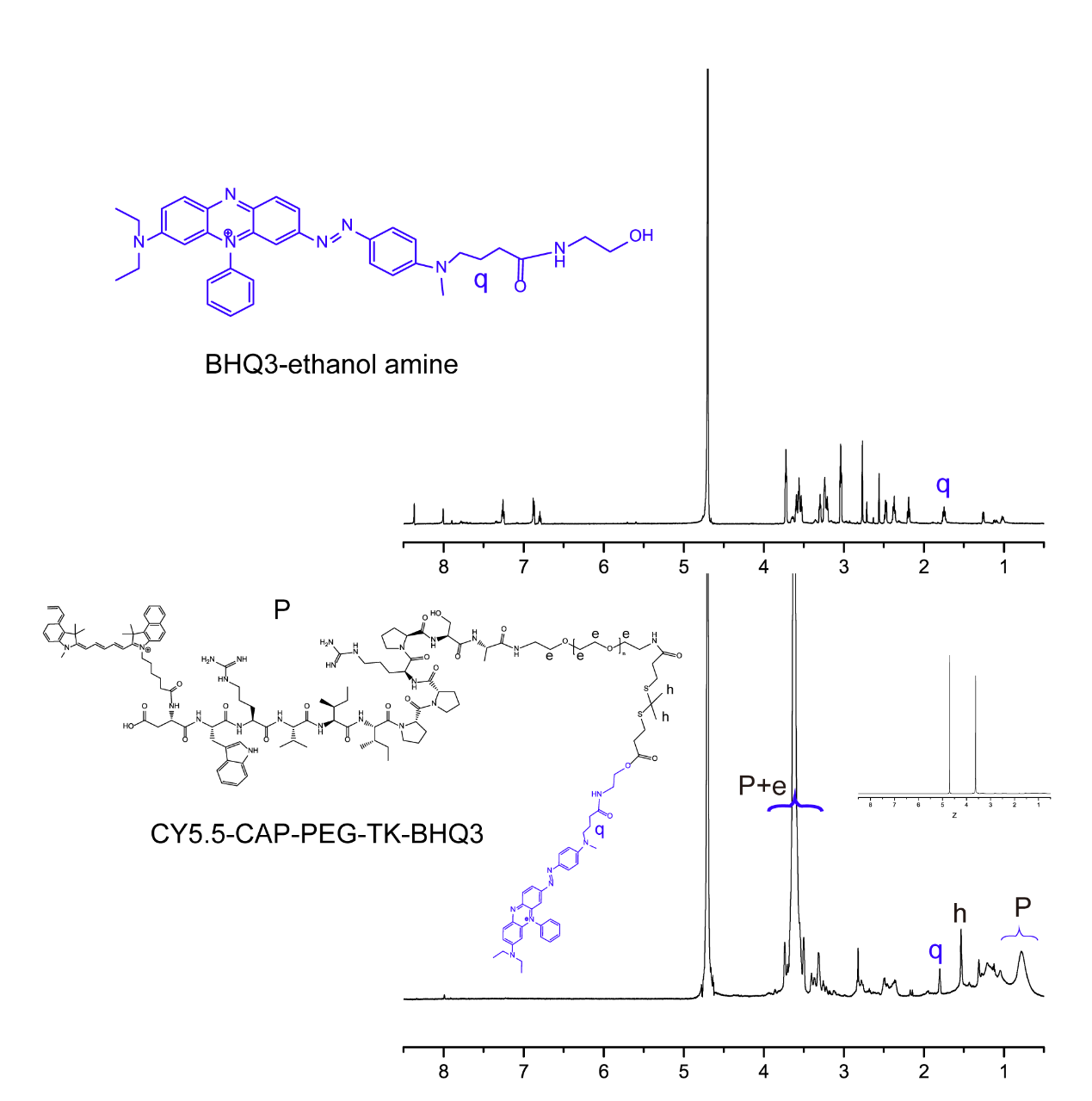


**Figure S9.** ^1^HNMR spectrum of Cy5.5-CAP-PEG-TK-BHQ3 in D_2_O.


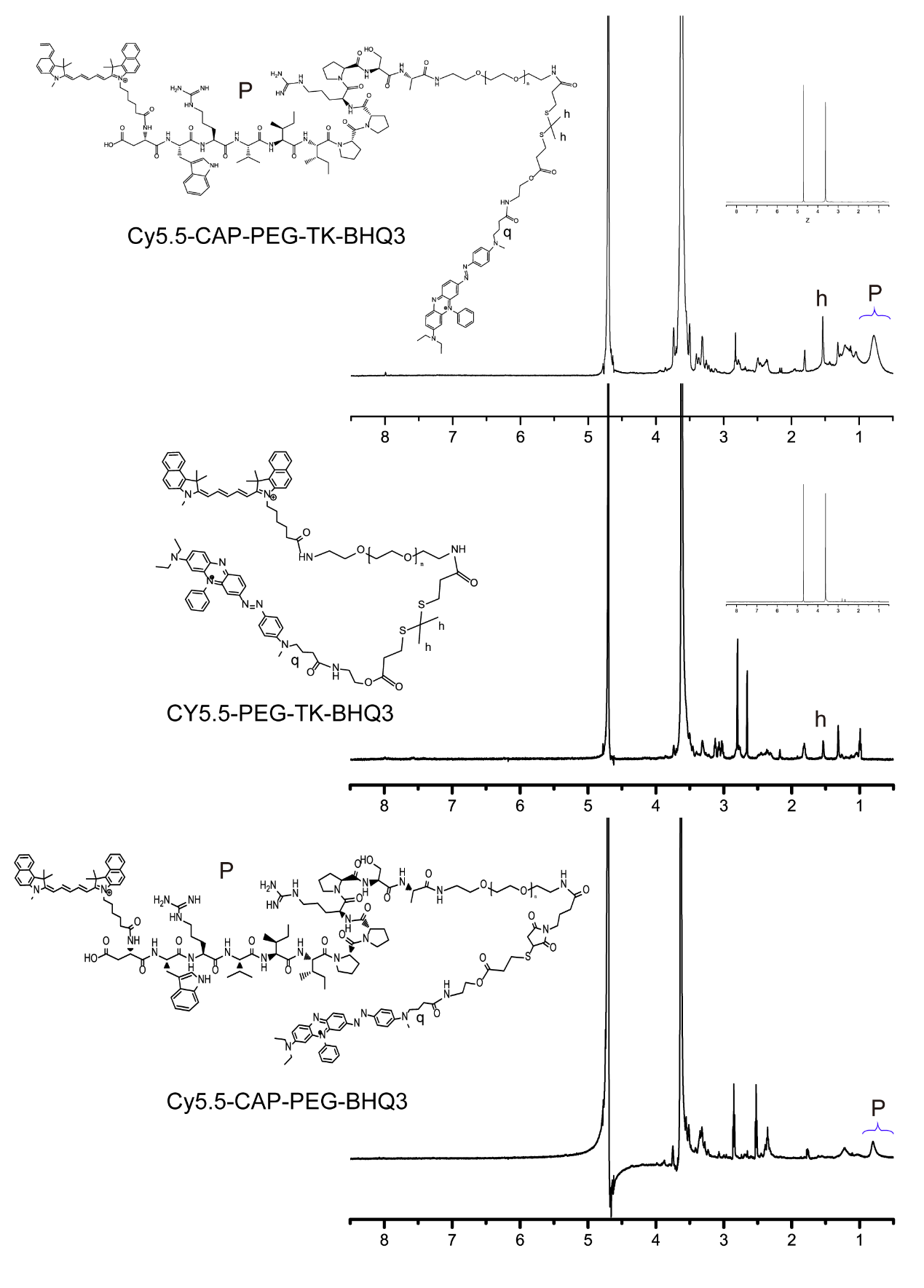


**Figure S10.** ^1^HNMR spectrum of Cy5.5-CAP-PEG-TK-BHQ3, Cy5.5-PEG-TK-BHQ3 and Cy 5.5-CAP-PEG-BHQ3 in D_2_O.
